# Histoid Leprosy

**DOI:** 10.4269/ajtmh.14-0658

**Published:** 2015-06-03

**Authors:** Kaliaperumal Karthikeyan

**Affiliations:** Department of Dermatology and STD, Sri Manakula Vinayagar Medical College and Hospital, Kalitheerthalkuppam, Puducherry, India

## Abstract

Histoid leprosy is rare type of lepromatous leprosy characterized by unique clinical, histopathological, and microbiological features. It is characterized by cutaneous and subcutaneous nodules. Histoid leprosy cases represent probable resistant bacilli and a highly active lepromatous process. These cases may act as reservoirs of the disease and lead to further spread of leprosy. Continual occurrence of these cases does not bode well for eradication of leprosy.

A 20-year-old young male, an agricultural laborer, complained of multiple asymptomatic shiny nodules all over the body. The lesions were present for the last 1 year. He had two episodes of epistaxis in the last 6 months that were treated symptomatically. The nodules were distributed predominantly over the trunk and thighs. Family history was non-contributory. He did not take any form of treatment for his skin lesions. Clinical examination revealed multiple discrete, skin-colored, shiny cutaneous and subcutaneous nodules over the trunk and limbs. The nodules varied in size from 0.5 to 1.0 cm (Figure 1

**Figure 1.**
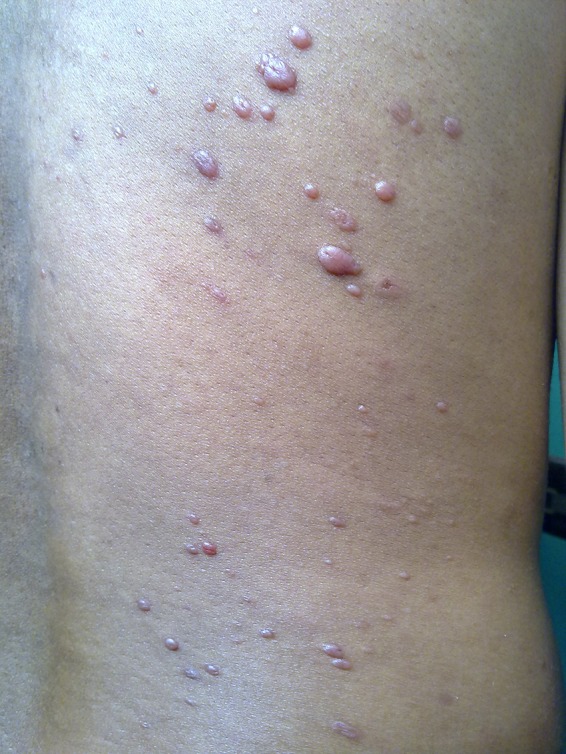
Multiple skin-colored nodules on the trunk.

); there was no impairment of pain, touch, or temperature sensation. There was no thickening of peripheral nerves. Slit skin smear from the nodule revealed plenty of acid fast bacilli, with a bacteriological index of 6+. Histopathology of the nodule showed atrophic epidermis with a subepidermal grenz zone. The dermis revealed sheets of spindle cells arranged in a whorled, crisscross/storiform pattern (Figure 2

**Figure 2.**
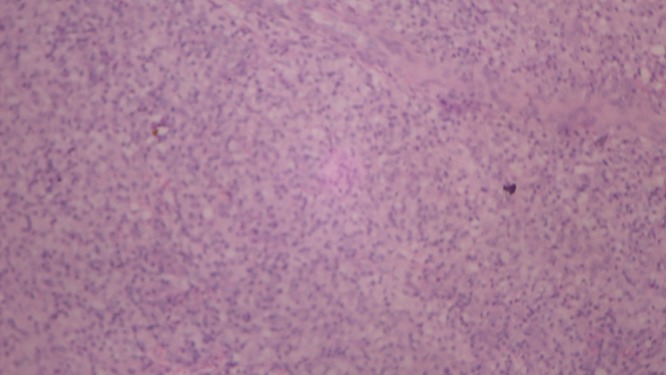
High-power (40×) view of the histopathology showing the spindle cells in a whorled pattern.

). Fite's stain showed numerous acid fast bacilli (Figure 3). The patient was diagnosed with the histoid variant of lepromatous leprosy. He was treated with multibacillary–multidrug therapy (MB-MDT) comprising of monthly rifampicin and daily dapsone and clofazimine for a period of 1 year. The patient responded well to the treatment.

**Figure 3.**
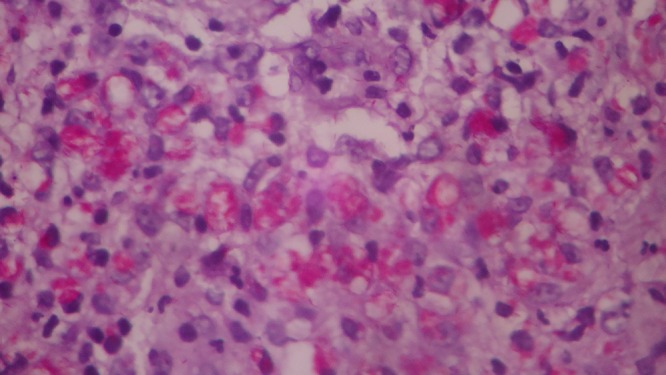
Oil immersion (100×) view of the Fite's stain showing the macrophages with acid fast bacilli.

Histoid leprosy is a distinct and rare variant of lepromatous leprosy, and it was described by Wade^1^ in 1963. It is characterized by unique clinical, histopathological, and microbiological features. This form of leprosy is fairly common in patients on dapsone monotherapy and irregular treatment.^2^ Sometimes, it can arise *de novo* as well. The clinical features include cutaneous and subcutaneous nodules and plaques. The nodules are well-demarcated, and the skin surrounding the lesions is apparently normal. The histopathological features are distinct and contribute to the nomenclature of the condition. The most prominent feature is the presence of numerous spindle-shaped histiocytes arranged in interlacing bands, whorls, and tight curlicues.^2^

Histoid leprosy cases represent probable resistant bacilli and a highly active lepromatous process. Continual occurrence of these cases does not bode well for a country like India, where leprosy was eliminated as a public health problem in 2005.^3^ These cases may act as reservoirs of the disease and lead to further spread of leprosy. Early diagnosis and management of these cases are important in this era of leprosy eradication.
